# Artificial consortium demonstrates emergent properties of enhanced cellulosic-sugar degradation and biofuel synthesis

**DOI:** 10.1038/s41522-020-00170-8

**Published:** 2020-12-02

**Authors:** Heejoon Park, Ayushi Patel, Kristopher A. Hunt, Michael A. Henson, Ross P. Carlson

**Affiliations:** 1 Department of Chemical and Biological Engineering, Montana State University, Bozeman, MT USA; 2 Center for Biofilm Engineering, Montana State University, Bozeman, MT USA; 3 Department of Chemical Engineering and Institute for Applied Life Sciences, University of Massachusetts, Amherst, MA USA; 4 Present Address: Department of Engineering and Technology, University of North Alabama, Florence, AL USA; 5 Present Address: Department of Civil and Environmental Engineering, University of Washington, Seattle, WA USA

**Keywords:** Applied microbiology, Biofilms, Microbial ecology

## Abstract

Planktonic cultures, of a rationally designed consortium, demonstrated emergent properties that exceeded the sums of monoculture properties, including a >200% increase in cellobiose catabolism, a >100% increase in glycerol catabolism, a >800% increase in ethanol production, and a >120% increase in biomass productivity. The consortium was designed to have a primary and secondary-resource specialist that used crossfeeding with a positive feedback mechanism, division of labor, and nutrient and energy transfer via necromass catabolism. The primary resource specialist was *Clostridium phytofermentans* (*a.k.a. Lachnoclostridium phytofermentans*), a cellulolytic, obligate anaerobe. The secondary-resource specialist was *Escherichia coli*, a versatile, facultative anaerobe, which can ferment glycerol and byproducts of cellobiose catabolism. The consortium also demonstrated emergent properties of enhanced biomass accumulation when grown as biofilms, which created high cell density communities with gradients of species along the vertical axis. Consortium biofilms were robust to oxic perturbations with *E. coli* consuming O_2_, creating an anoxic environment for *C. phytofermentans*. Anoxic/oxic cycling further enhanced biomass productivity of the biofilm consortium, increasing biomass accumulation ~250% over the sum of the monoculture biofilms. Consortium emergent properties were credited to several synergistic mechanisms. *E. coli* consumed inhibitory byproducts from cellobiose catabolism, driving higher *C. phytofermentans* growth and higher cellulolytic enzyme production, which in turn provided more substrate for *E. coli*. *E. coli* necromass enhanced *C. phytofermentans* growth while *C. phytofermentans* necromass aided *E. coli* growth via the release of peptides and amino acids, respectively. In aggregate, temporal cycling of necromass constituents increased flux of cellulose-derived resources through the consortium. The study establishes a consortia-based, bioprocessing strategy built on naturally occurring interactions for improved conversion of cellulose-derived sugars into bioproducts.

## Introduction

Sustainable, cost-effective production of fuels and chemicals is a major societal challenge. Lignocellulosic biomass is a promising feedstock for bioprocesses because of the large global supply, low cost, and the flexibility of the monomers to be converted into value-added products, including fuels, chemicals, and materials^1,2^. Consolidated, one pot, bioprocessing where lignocellulose depolymerization and product formation occur in a single vessel, is proposed to be a cost-effective strategy for producing fuels and chemicals due to process simplicity^3,4^. Biological routes for lignocellulose depolymerization are environmentally and economically attractive due to high substrate conversion and mild operating conditions as compared to the high energy and harsh chemical requirements of thermochemical processes^5^.

Traditional bioprocessing efforts have focused primarily on using a single “superbug” to achieve all desired chemistries. However, using single organisms for consolidated bioprocess often leads to low product titers, yields, and productivities^6–9^. It is difficult to optimize all necessary traits simultaneously due to tradeoffs in resource allocation^10^. Resources allocated to one function are not available to optimize additional functions; this concept forms the basis of the “Darwinian Demon” ecological thought experiment^11,12^. Evolution and natural selection have addressed the challenge of complex, multistep processes, like lignocellulose deconstruction via consortia using division of labor^8,13–18^. Natural and assembled consortia have been used for degrading lignocellulosic substrates^8,19–24^. The assembled consortia have used combinations of fungi or fungi and bacteria. For example, Minty et al.^25^ have used *Escherichia coli* and *Trichoderma reesei* to produce isobutanol from cellulose while Jin et al.^23^ and Zuroff et al.^21^ have assembled consortia comprised of *Clostridium phytofermentans* (*a.k.a. Lachnoclostridium phytofermentans*) and *Saccharomyces cerevisiae* to produce ethanol from cellulosic feedstocks.

Biofilms are microbial aggregates encapusulated in self-produced polymers and are typically associated with an interface like a solid surface; in nature, most microorganisms reside in biofilms^26^. The biofilm phenotype is distinct from the planktonic phenotype. Rate imbalances between biotic reactions and abiotic diffusion create gradients in chemicals and metabolic activity. These gradients are largely responsible for the structure and physiology of biofilms and can be viewed as control parameters for bioprocess applications^27–29^. Biofilms have competitive properties for bioprocessing including high cell densities (200–300 g cell dry weight L^−1^), high volumetric productivities, reduced requirements for water, no need for energy intensive agitation, facilitated separation of biomass from supernatant, and high tolerance to stresses like pH or inhibitors^10,30,31^.

There is considerable scientific interest in improving the catalytic efficiency of natural processes like nutrient cycling and applied processes like biofuel synthesis. Harnessing the emergent properties of microbial interactions has the potential to achieve this catalytic goal^9^. However, the biological compentents and interactions necessary to achieve emergent properties are not well understood. Natural systems are often extremely complex in terms of the number of species and the number of interactions, confounding the basis of emergent properties. Synthetic and artifical ecology have ability to decode the requirements of nonlinear, emergent properties^15^. In this work, an artificial consortium comprised of *C. phytofermentans* and *E. coli* was constructed. Here, the term artifical consortium is used to describe a consortium comprised of wild-type organisms that are not thought to cooccur in nature; alternatively, a synthetic consortium is defined as a consortium with at least one genetically modified population^32^. *C. phytofermentans* is a mesophilic, obligate anaerobe that grows on both soluble and insoluble components of lignocellulosic feedstocks^33^. *C. phytofermentans* is remarkable among the *Clostridium* genus due to its ability to catabolize a broad range of substrates. Its genome encodes over 169 carbohydrate-active enzymes, the largest number among sequenced clostridia, and its efficient ethanol production makes it a model system for cellulosic biofuel production^21,23,34–37^. *E. coli* is a well studied, facultative anaerobe capable of fermenting a broad range of substrates including glucose and glycerol which is a widely available waste product from biodiesel production^21,23,38^. *E. coli* is also a convenient host for metabolic engineering and can be modified to produce a wide range of biochemical products^39,40^. The *C. phytofermentans* and *E. coli* consortium was assembled to leverage common ecological motifs including cooccurrence of primary and secondary-resource specialists, metabolite exchange with positive feedback, and the flux of nutrients and energy between trophic levels through the catabolism of lysed biomass known as necromass^27,41–46^. Additionally, when grown as a biofilm, *E. coli* consumes O_2_ creating an anoxic environment for *C. phytofermentans*. The role of each consortium member, the mechanisms of interaction, and the spatial and temporal analysis of system function were considered in this study quantifying the enhanced consortium productivity. The metrics used to quantify the emergent properties of the consortium were (1) enhanced depletion of cellulosic sugar, (2) enhanced production of ethanol as a proxy biofuel and bioproduct molecule, and (3) enhanced production of microbial biomass.

## Results

### Planktonic monocultures and consortium properties

**Fig. 1:**
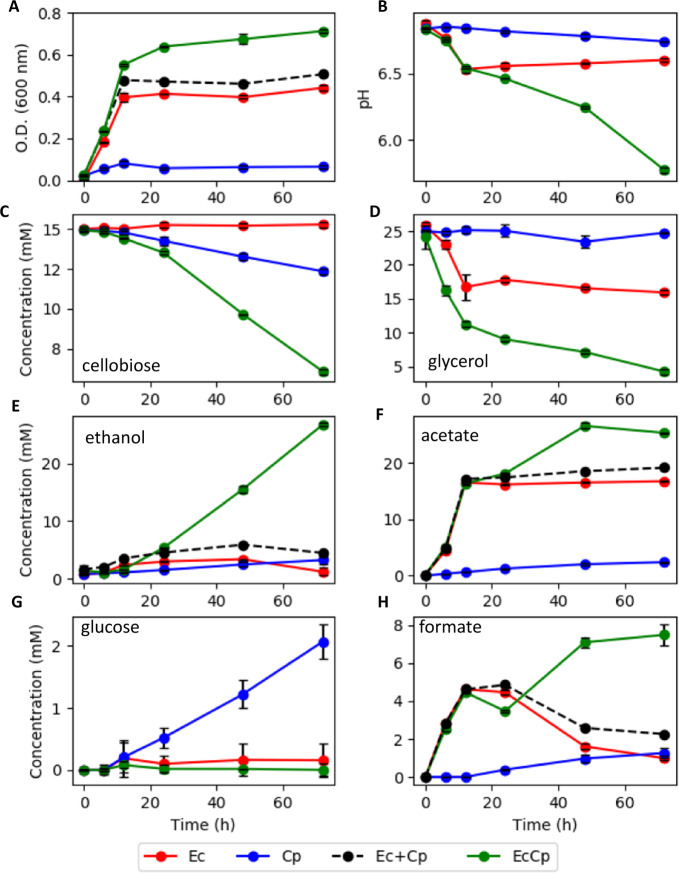
Planktonic growth properties for *E. coli* (Ec) and *C. phytofermentans* (Cp) monocultures and a binary consortium (EcCp) under anoxic conditions. **a** Optical density (OD_600_), **b** pH, **c** cellobiose concentration, **d** glycerol concentration, **e** ethanol concentration, **f** acetate concentration, **g** glucose concentration, and **h** formate concentration. Ec + Cp indicates the sum of *E. coli* and *C. phytofermentans* monoculture properties. Trends are only shown where relevant. All experiments were performed using mGS-2 medium. Error bars represent the standard deviation from three biological replicates.

A consortium of *C. phytofermentans* and *E. coli* was assembled based on compatible physiologies, culturing conditions, and the possibility of synergistic interactions. The consortium was characterized as an anoxic planktonic culture to facilitate analysis of phenotypes, consortium member roles, and intercellular metabolite exchanges. The consortium demonstrated the emergent properties of enhanced substrate depletion, enhanced ethanol secretion, and enhanced biomass production as compared to monocultures (Fig. 1a, c, e and Table 1). The consortium consumed 8.84 ± 0.06 mM of cellobiose over 72 h of cultivation which was a 240% increase over *C. phytofermentans* monocultures (2.60 ± 0.11 mM). *E. coli* monocultures did not catabolize cellobiose, as expected; *E. coli* does not possess a functional cellobiase^47^. *C. phytofermentans* monocultures accumulated glucose during batch growth while free glucose was not measured during consortium cultivation, presumably due to rapid catabolism of the monosaccharide by *E. coli*. *E. coli* monocultures fermented 9.85 ± 0.89 mM of glycerol over 72 h while *C. phytofermentans* monocultures did not catabolize glycerol (Fig. 1d). *E. coli* catabolism of glycerol doubled to 19.89 ± 1.33 mM under consortium cultivation. mGS-2 medium contained citrate as an ion chelator; *E. coli* readily fermented citrate in the presence of glycerol while *C. phytofermentans* did not oxidize citrate (Supplementary Table 1 and Supplementary Fig. 1A). The observed, emergent properties were robust to changes in culture medium. Four different formulations of mGS-2 medium were analyzed; all resulted in similar, enhanced cellobiose (Fig. 1c), ethanol (Fig. 1e), and biomass properties (Fig. 1a and Supplementary Figs. 2–4).

**Table 1:** Summary of growth parameters compared between planktonic monocultures of *E. coli* (Ec) and *C. phytofermentans* (Cp) and a planktonic, binary consortium.

	Ec	Cp	Ec + Cp (A)	Binary (B)	Δ (B−A)	Increase (%)
Cellobiose (mM)	–	−2.60 ± 0.11	−2.60	−8.84 ± 0.06	−6.24	240
Ethanol (mM)	0.44 ± 0.64	2.41 ± 0.47	2.85	26.71 ± 0.61	23.86	837
Acetate (mM)	16.66 ± 0.07	2.41 ± 0.11	19.07	25.40 ± 0.02	6.33	33
Formate (mM)	0.99 ± 0.11	1.27 ± 0.24	2.26	7.49 ± 0.54	5.23	231
Glycerol (mM)	−9.85 ± 0.89	–	−9.85	−19.89 ± 1.33	−10.04	102
Biomass (g/L)	0.090 ± 0.028	0.055 ± 0.021	0.145	0.315 ± 0.007	0.175	121

Ec + Cp is the sum of monoculture properties. Data collected after 72 h of cultivation. Error represents standard deviation from three biological replicates. Δ = difference between culture samples.

Increased catabolism of cellobiose and glycerol resulted in higher titers of byproducts. The consortium produced 26.71 ± 0.61 mM ethanol, 25.40 ± 0.02 mM acetate, and 7.49 ± 0.54 mM formate (Table 1). Cultures produced small (<1.2 mM), but measurable, amounts of lactate (Supplementary Fig. 1B); no succinate was observed. Consortium pH values where lower than the monocultures reflecting the increased catabolism of cellobiose and glycerol and increased secretion of acidic byproducts. Consortium pH dropped to 5.8 over the course of 72 h while the *C. phytofermentans* and *E. coli* monoculture pH dropped to 6.7 and 6.6, respectively (Fig. 1b).

*E. coli* cultures accumulated biomass for approximately 12 h while *C. phytofermentans* cultures also accumulated biomass for 12 h but continued to catabolize substrate and secrete byproducts for 72 h (Fig. 1a, c, e). The consortium had a 41% increase in optical density (OD_600_) relative to the sum of the monocultures. Biomass productivity of the consortium was substantially larger than the sum of monoculture cell dry weights, increasing 121% (Table 1). Quantitative relationships between OD_600_, colony-forming units (CFU) per liter and gram cell dry weight per liter can be found in the materials and methods.

### Biofilm phenotypes of consortium, with culturing perturbations

**Fig. 2:**
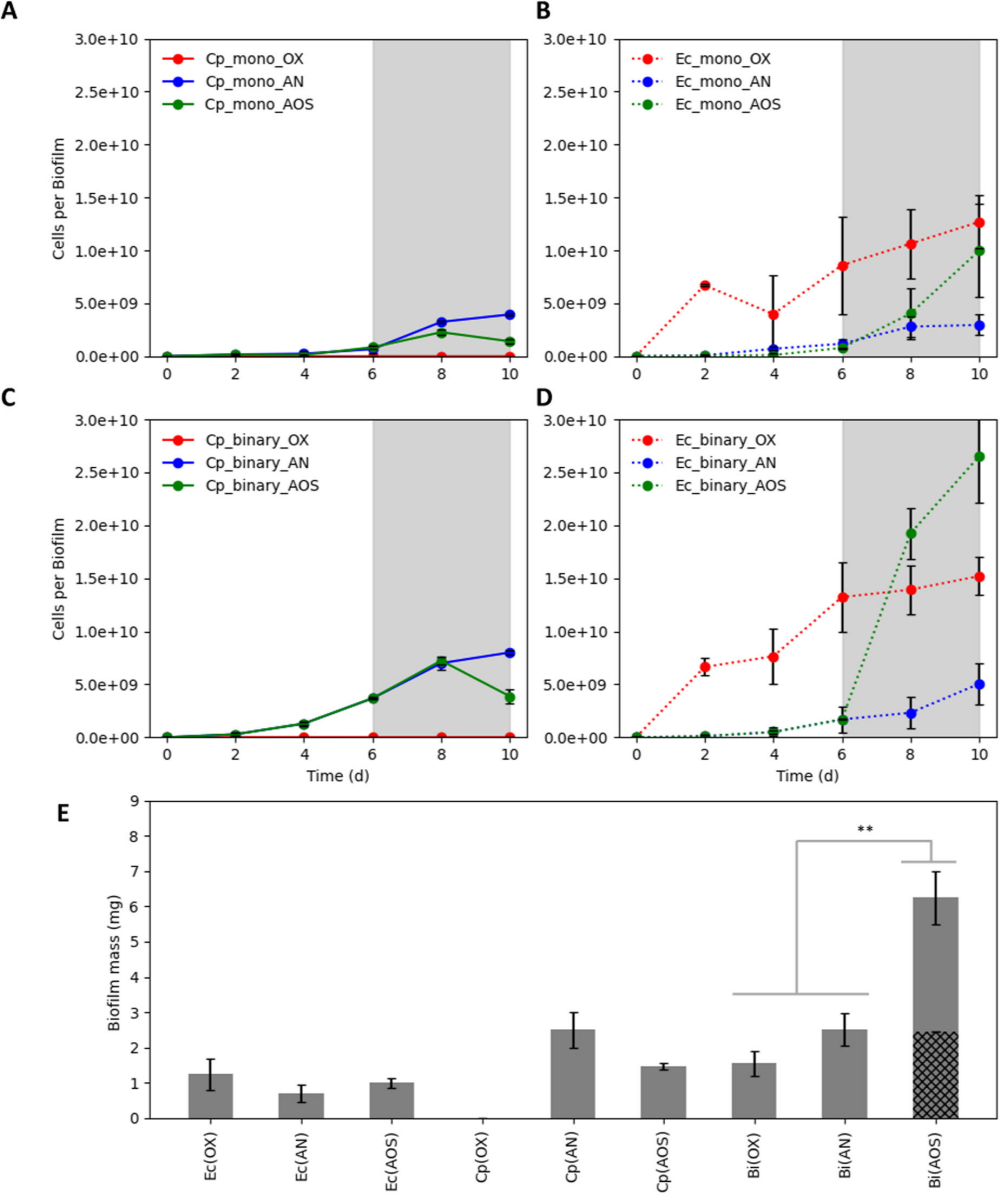
Biofilm biomass productivity for *E. coli* (Ec) and *C. phytofermentans* (Cp) monocultures and binary consortium (Bi) grown under three different cultivation conditions. OX: 10 days of oxic only conditions, AN: 10 days of anoxic only conditions, and AOS: 6 days anoxic and 4 days oxic growth (denoted with gray shading). **a** *C. phytofermentans* cell number per monoculture biofilm, **b** *E. coli* cell number per monoculture biofilm, **c** *C. phytofermentans* cell number per consortium biofilm, **d** *E. coli* cell number per consortium biofilm, **e** Total biofilm mass (biomass + extracellular material) for monoculture and consortium biofilm cultures. Black hashed area represents sum of monoculture data for AOS condition. Data in **e** collected after 10 days of cultivation. Error bars represent the standard deviation from three biological replicates. Statistical significance at **p < 0.01, *T*-test.

*C. phytofermentans* and *E. coli* monocultures and the consortium were grown as biofilms, a common, naturally occurring, growth state and potentially useful phenotype for bioprocesses^31,48^. Biofilm cultures were grown for 10 days using one of three cultivation strategies: completely anoxic, completely oxic, or an anoxic to oxic switch (AOS) after 6 days of cultivation (Fig. 2a–d). The AOS strategy was designed to quantify the robustness of the consortium to perturbations and to induce O_2_-based, lysis of *C. phytofermentans* cells to produce necromass.

Monoculture biofilms of *C. phytofermentans* grew under anoxic conditions and during the anoxic phase of the AOS cultivation (Fig. 2a). There was no biomass accumulation when the monoculture biofilms were incubated for 10 days in the presence of O_2_ (Fig. 2a). The AOS cultures had a decrease in cell number, based on qPCR, after 2 days of oxic cultivation. The initial increase in cell number during oxic culturing likely reflected growth prior to O_2_-based lysis. The cell number data were based on the presence of chromosomal DNA and not necessarily viable cells.

*E. coli* monoculture biofilms produced more biomass under oxic conditions relative to anoxic, as anticipated. The presence of O_2_ made more substrate energy bioavailable (Fig. 2b). The effect of O_2_ was especially apparent during AOS cultivation; the introduction of O_2_ after 6 days resulted in rapid, biomass accumulation as the fermentation byproducts like acetate and nonfermentable amino acids found in the medium were likely oxidized aerobically (Fig. 2b).

Anoxic and AOS consortium growth increased the biomass productivity of both *C. phytofermentans* and *E. coli* relative to monoculture biofilms (Fig. 2c, d). The enhanced growth also implied an enhanced use of available substrates. *C. phytofermentans* grown as a consortium biofilm more than doubled biomass productivity as compared to the monoculture biofilm (Fig. 2a, c). During AOS cultivation, *C. phytofermentans* cell number, as detected by qPCR, increased initially following transition to oxic conditions before decreasing. The time delay, before the cell number decreased, could have been a result of O_2_ diffusion, cell lysis, and DNA degradation kinetics. The largest increase in *E. coli* biomass accumulation occurred during cultivation of the consortium under AOS conditions (Fig. 2b, d). There was a rapid increase in *E. coli* biomass upon switching to an oxic environment where accumulated fermentation byproducts could be catabolized and additionally, the O_2_-lysed *C. phytofermentans* biomass was available for *E. coli* catabolism (Fig. 2d). *E. coli* growth under AOS conditions exceeded *E. coli* growth under continuous O_2_ exposure, quantifying how the temporal partitioning of metabolism can enhance culture performance.

The consortium, grown under completely oxic conditions, did not show enhanced biomass productivity. *C. phytofermentans* was inhibited by O_2_ at inoculation, leading to a biofilm that was functionally, an *E. coli* monoculture (Fig. 2b–d).

Biofilm productivity was also analyzed using direct, gravimetric analysis on day 10 (Fig. 2e). *C. phytofermentans* and *E. coli* have different cellular geometries and, therefore, a comparison of cell number does not reflect total cell mass. Additionally, qPCR quantifies copy number of DNA sequences and would not quantify the production of other biofilm components like extracellular polymeric substance (EPS). *C. phytofermentans* monoculture biofilms produced over 2 mg cellular material (biomass and EPS) per biofilm during anoxic cultivation; no biomass accumulation was observed under oxic conditions and the AOS condition had an intermediate mass of cellular material. *E. coli* monoculture biofilms had a mass of 0.7–1.2 mg cellular material per biofilm depending on cultivation strategy; AOS and oxic cultivation produced the larger masses. AOS cultivation resulted in a large increase in consortium biomass. AOS consortium accumulated 6.25 mg cellular material per biofilm which was 153% more material than the sum of the *E. coli* and *C. phytofermentans* monocultures grown under AOS conditions (Table 2).

**Table 2:** Biofilm productivity expressed as mass of total cellular material (biomass + polymeric material) produced in 10 days for monocultures of *E. coli* (Ec) and *C. phytofermentans* (Cp) and consortium biofilms (EcCp). Ec + Cp is the sum of monoculture properties.

	Ec (AOS)	Cp (AOS)	Ec + Cp (A)	EcCp (AN) (B)	EcCp (AOS) (C)	Δ (C−A)	Δ (C−B)
Biofilm mass (g)	1.00 ± 0.13	1.47 ± 0.09	2.47	2.53 ± 0.46	6.25 ± 0.75	3.78 (+153%)	3.72 (+147%)

AN: 10 days of anoxic only conditions, AOS: 6 days anoxic and 4 days oxic growth. Biofilm masses were collected after 10 days of culturing. Data are from three biological replicates. Δ = difference between culture samples, A, B, and C.

### Spatially resolved analysis of biofilm cultures

**Fig. 3:**
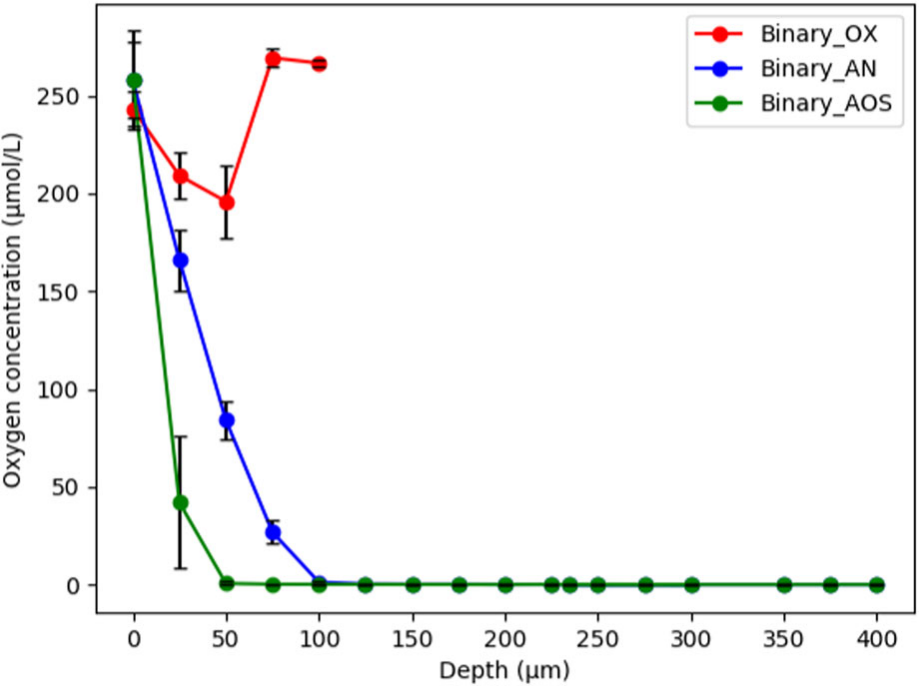
Spatially resolved, in situ, O_2_ concentration in *E. coli* and *C. phytofermentans* consortium biofilms grown using three different cultivation strategies. OX: grown for 10 days oxically, AN: grown for 10 days anoxically, AOS: grown for 6 days anoxically followed by 4 days of oxic growth. The O_2_ concentrations within biofilm were measured using 25 µm diameter microelectrode O_2_ probes. A depth of 0 μm is the top surface of the biofilm. Error bars represent the standard deviation from three biological replicates.

**Fig. 4:**
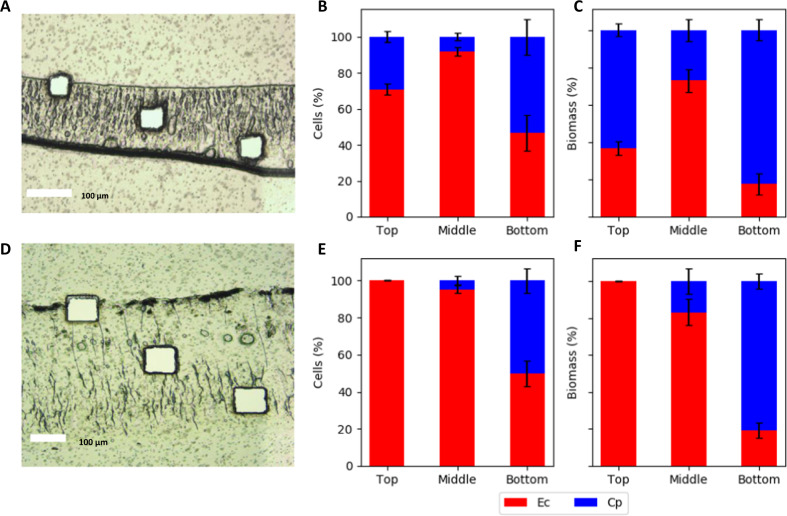
Spatially-resolved, species distributions in *E. coli* (Ec) and *C. phytofermentans* (Cp) consortium biofilms. **a**–**c** Consortium biofilms grown anoxically (AN) for 10 days and **d**–**f** consortium biofilms grown anoxically for 6 days followed by 4 days of oxic growth (AOX). Species distributions were measured using laser microdissection and qPCR analysis of 16S gene copy number. Biomass percentage was calculated from cell number data converted to mass using conversion factors listed in the “Materials and methods”. Cell data based on biofilm samples taken from three vertical positions (top, middle, bottom) and four to six radial positions from a single biofilm. Error bars represent the standard deviation of samples. Micrograph scale bars = 100 μm.

Spatially resolved in situ O_2_ concentrations were measured within the biofilms on day 10 (Fig. 3). The AOS cultivation strategy produced the thickest biofilm (275–475 µm) (Figs. 3 and 4d and Supplementary Fig. 5); the in situ O_2_ concentration was below detection 50 µm from the oxic interface, creating a large anoxic zone for *C. phytofermentans*. The oxic conditions produced the thinnest biofilms (17–34 µm) which were oxic from top to bottom (≥75% of saturation) (Fig. 3 and Supplementary Fig. 5). The consortium biofilms, cultivated for 10 days anoxically, consumed O_2_ as soon as they were removed from the anoxic incubator and reduced O_2_ concentrations below detection within 100 µm from the oxic surface (Fig. 3) (it took ~15 min to remove the biofilms from the incubator, to transport the biofilms to the microelectrode equipment, and to make the O_2_ measurements). This rapid response indicated the *E. coli* had the enzymatic machinery to respire O_2_ expressed, even though the cultures were not exposed to the electron acceptor for 10 days. As a reference calculation, the abiotic diffusion of O_2_ through a 150–200 µm biofilm (Fig. 4d) would be predicted to take approximately 14–25 s, assuming the effective diffusion coefficient of O_2_ within a biofilm was 8 × 10^−6^ cm^2^ s^−1^ ^49^ and assuming there were no O_2_ consuming reactions. Therefore, the observed O_2_ profiles reflected biological consumption and not solely a diffusion process.

The spatial distributions of species and cell concentrations were measured using a combination of biofilm cryosectioning, laser microdissection, and qPCR. Samples were collected from three vertical locations: top, middle, and bottom of the biofilms at four to six radial positions (Fig. 4a, d). During anoxic cultivation, *C. phytofermentans* accounted for ~30% of the total cell number, based on qPCR, and ~70% of the total cell mass at the top and bottom of the biofilm. *E. coli* accounted for >70% of the total cell number and cell mass in the middle section of the biofilm, suggesting an optimal, spatial environment where glucose and *C. phytofermentans* necromass were available (Fig. 4b, c).

AOS cultivation showed different results. First, the biofilms were more than twofold thicker than the anoxic biofilm based on the cryosectioned samples (Fig. 4a, d) and optical coherence tomography analysis of hydrated biofilms (Supplementary Fig. 5). Second, *C. phytofermentans* resided primarily in the anoxic bottom of the biofilm, where it represented ~55% of the total cell number and ~84% of the total cell mass (Fig. 4e, f). *E. coli* comprised more than 99% of the total cell number and total cell mass at the top, oxic layer of the biofilm, and >75 % of the total cell number and cell mass in the middle of the biofilm.

**Fig. 5:**
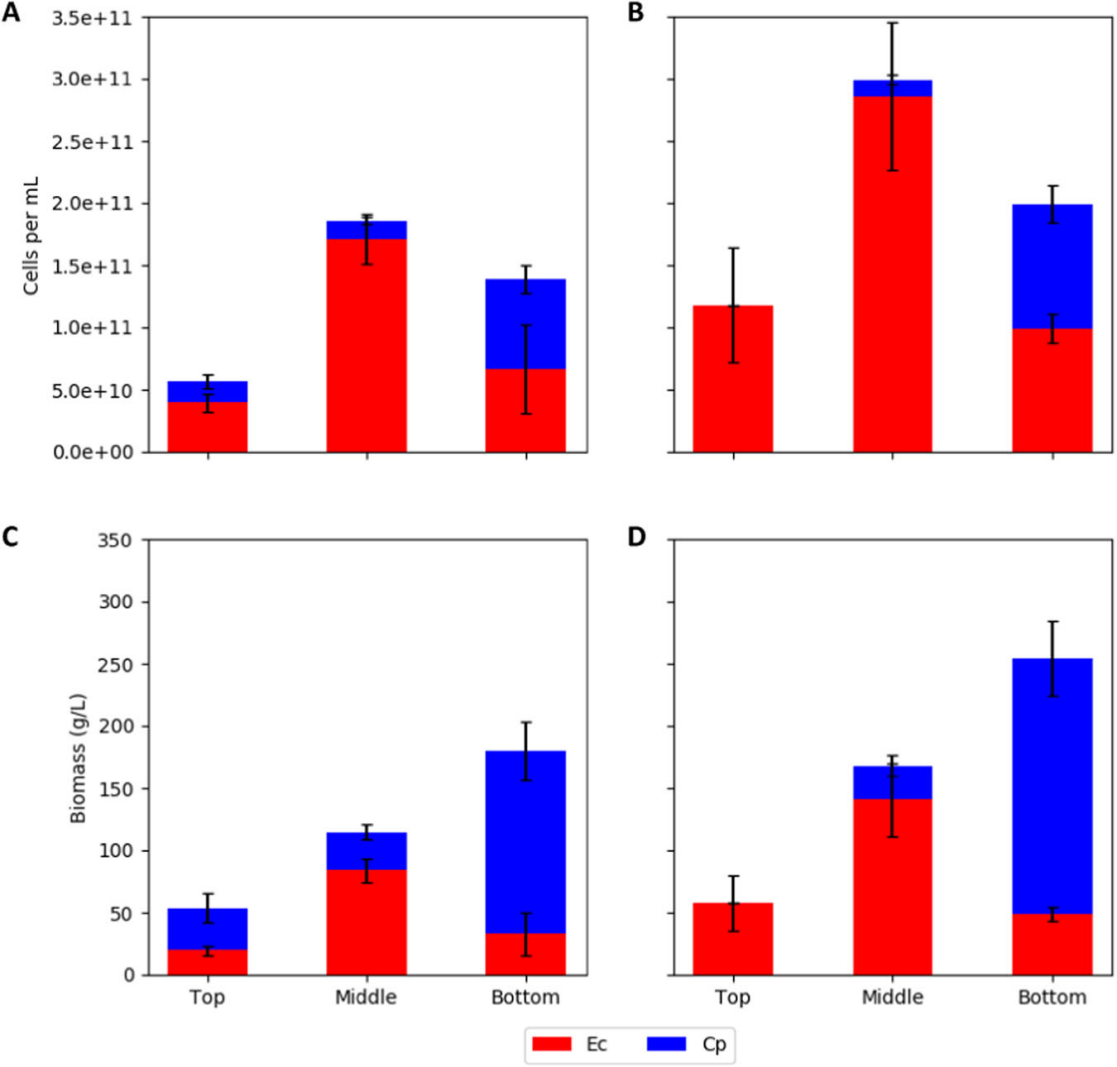
*E. coli* (Ec) and *C. phytofermentans* (Cp) cell number and biomass concentration as a function of spatial locations in consortium biofilms. **a**, **c** Consortium biofilms grown for 10 days anoxically (AN) and **b**, **d** consortium biofilms grown anoxically for 6 days followed by 4 days of oxic growth (AOS). Data are from day 10. Cryosectioned biofilms had cells samples excised using laser microdissection from three vertical positions (top, middle, bottom) from four to six radial positions. Cell number was calculated using qPCR. Biomass concentrations were calculated using conversion factors listed in “Materials and methods”. Error bars represent the standard deviation of samples.

The cellular distributions provided data for calculating the total cell number and total cellular mass as a function of spatial position in the biofilm (Fig. 5a–e). The total cell number peaked in the middle of the biofilm for both the AN and AOS biofilms, reaching approximately 2–2.5 × 10^11^ cells per mL of biofilm. The cellular mass concentration was highest at the bottom of the biofilm with densities of 0.25–0.30 g biomass per mL.

### Mechanisms of enhanced consortium performance: role of cellobiase and glucose inhibition

**Fig. 6:**
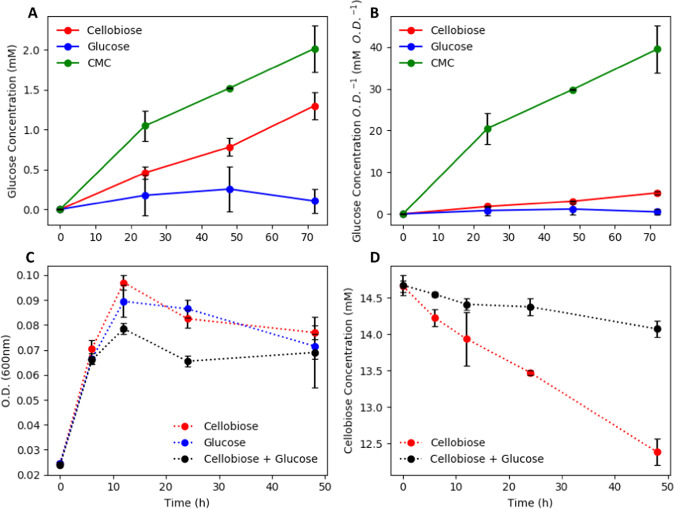
Cellobiase activity (cellobiose hydrolysis to glucose) in spent medium from *C. phytofermentans* (Cp) cultures grown on various carbon sources (5 g L^−1^ of glucose, cellobiose, or CMC) and *C. phytofermentans* growth (OD_600_) with different carbon sources. **a** Volumetric cellobiase activity represented as liberated glucose concentration plotted as a function of time, **b** specific cellobiase activity represented as liberated glucose concentration normalized to culture OD_600_ plotted as a function of time, **c** *C. phytofermentans* growth (OD_600_) on cellobiose (5 g L^−1^), glucose (5 g L^−1^), and a mixture of sugars (5 g L^−1^ each), **d** Cellobiose consumption in *C. phytofermentans* monocultures with and without the presence of glucose (5 g L^−1^). Error bars represent the standard deviation from three biological replicates.

Possible mechanisms responsible for enhanced consortium performance were tested including the role of product inhibition on cellobiose degradation. Many cellulose degradation processes are inhibited, at either an enzyme activity- or regulation level, by the accumulation of degradation products such as glucose^50,51^. Planktonic, *C. phytofermentans* monocultures accumulated glucose during growth on cellobiose suggesting the release of cellobiase into the medium (Fig. 1g). Culture supernatants were collected during the stationary phase from *C. phytofermentans* monocultures grown on mGS-2 medium supplemented with either glucose (5 g L^−1^), cellobiose (5 g L^−1^), or carboxymethyl cellulose (CMC) (5 g L^−1^). Samples were filtered through 0.2 µm pore membranes to remove cells. Fresh cellobiose (5 g L^−1^) was added to the filtered supernatants and glucose production was monitored to measure cellobiase activity (Fig. 6a).

*C. phytofermentans* monocultures grown on CMC had the highest volumetric, cellobiase activity followed by the cellobiose- and the glucose-grown monocultures. This trend was further emphasized when the cellobiase activity was analyzed on a specific basis (volumetric activity normalized to culture OD_600_). CMC-grown monocultures had ~eight-fold higher specific cellobiase activity than the cellobiose-grown cultures (Fig. 6b). Monocultures grown on glucose containing medium did not produce statistically significant cellobiase activity. Enzyme activity was stable in the presence of O_2_; the cellobiase assays were performed under oxic conditions for 72 h.

The copresence of glucose and cellobiose negatively affected *C. phytofermentans* biomass accumulation and the degradation of cellobiose (Fig. 6c, d). This property was based on reduced production of cellulolytic enzymes (Fig. 6b) and likely due to some uncharacterized, catabolite repression mechanism. The *C. phytofermentans* genome contains three, annotated cellobiase/β-glucosidase genes. A candidate gene (ABX42305) for the *C. phytofermentans* cellobiase activity was identified based on similar extracellular activity, similar substrate repression, and the protein sequence alignment with enzyme BglA (AAQ00997) from *Clostridium cellulovorans*^52^. An alignment of the *C. phytofermentans* enzyme with the *C. cellulovorans* enzyme had 96% protein coverage, 31.4% protein identity, and an *E-*value of 5e^−56^.

### Mechanism of enhanced consortium performance: catabolism of *C. phytofermentans* necromass

The catabolism of *C. phytofermentans* necromass by *E. coli* was evaluated as another potential mechanism driving enhanced consortium performance. Necromass refers to released biomass components including macromolecules and free metabolites from lysed cells^21,53^. This cellular material could have served as a substrate for *E. coli*.

**Fig. 7:**
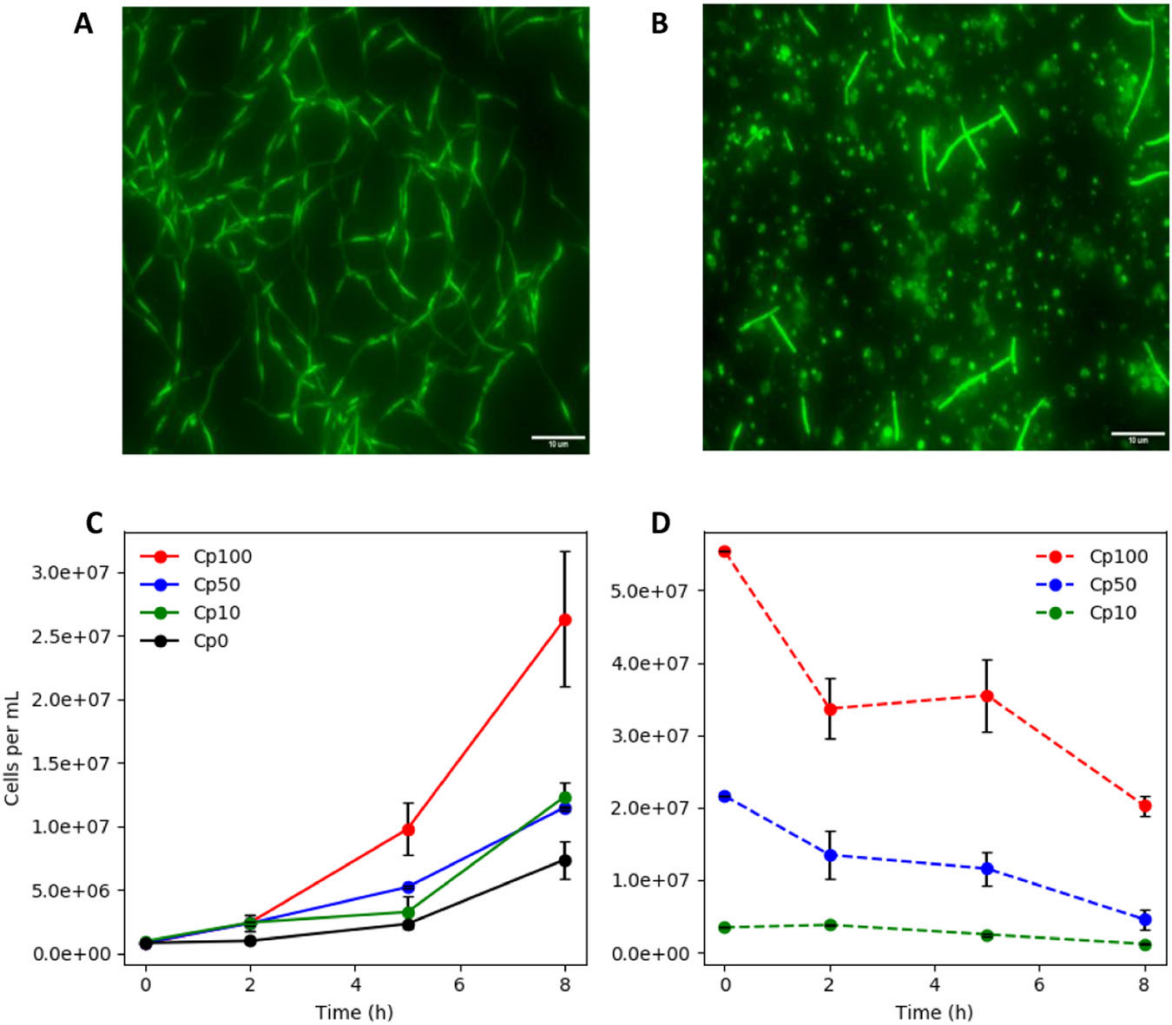
*E. coli* growth on *C. phytofermentans* (Cp) necromass. **a** Epifluorescence micrograph of *C. phytofermentans* cultured anoxically. **b** Epifluorescence image of lysed *C. phytofermentans* after 24 h of ambient air exposure. **c** Aerobic *E. coli* growth on different amounts of *C. phytofermentans* necromass, see main text for details. **d** *C. phytofermentans* necromass abundance, expressed as qPCR-based cell number, during aerobic *E. coli* growth on lysed *C. phytofermentans* biomass. Cp100, Cp50, Cp10, and Cp0 refer the percentage of medium comprised of *C. phytofermentans* necromass solution, see text for more details. Error bars represent the standard deviation from three biological replicates. Micrograph scale bars = 10 μm.

*C. phytofermentans* necromass was produced from monocultures, grown anoxically to mid-exponential phase. The cultures were harvested by centrifugation, washed in M9 medium^54^ with no carbon source, and then exposed to ambient air for 24 h to induce cell lysis. *C. phytofermentans* readily lysed in the presence of O_2_, as documented with microscopy (Fig. 7a, b). *E. coli* growth on *C. phytofermentans* necromass was tested under oxic conditions. Different concentrations of *C. phytofermentans* necromass were added to M9 minimal medium as the sole carbon source (Fig. 7c). *E. coli* produced more biomass, as quantified using qPCR, with increasing concentrations of necromass. The control *E. coli* culture, with no added *C. phytofermentans* necromass, showed an increase in DNA, likely due to cellular division based on storage compounds like polyglucose. The abundance of *C. phytofermentans* DNA, as quantified by qPCR, decreased with time potentially due to abiotic DNA degradation similar to environmental DNA degradation or due to released DNase enzymes (Fig. 7d)^55–58^.

**Fig. 8:**
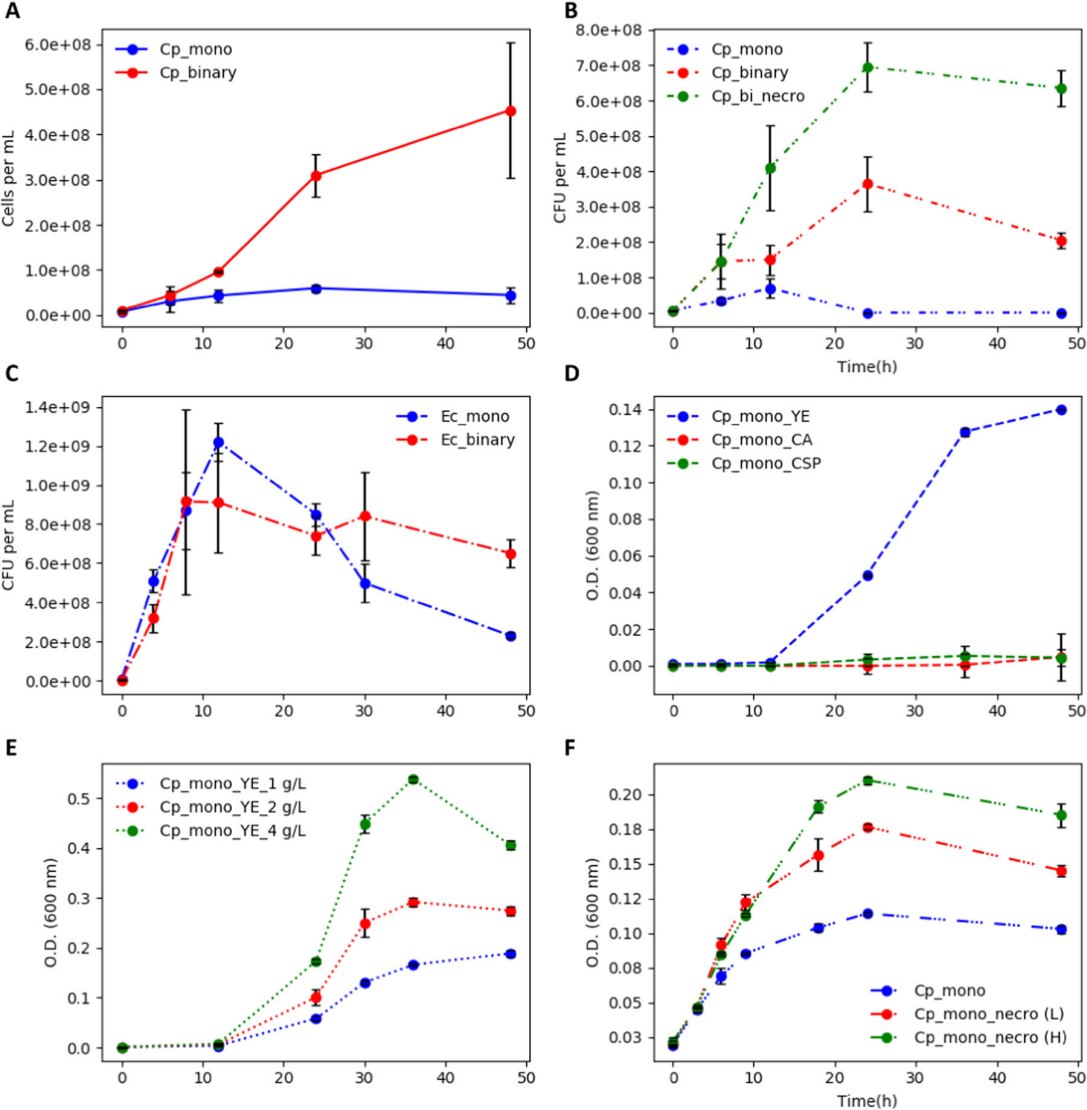
*C. phytofermentans* (Cp) growth on *E. coli* (Ec) necromass. **a** *C. phytofermentans* cell concentration during monoculture and binary consortium growth, based on qPCR analysis. **b** *C. phytofermentans* growth as a monoculture or binary consortia. Consortia growth tested both with and without *E. coli* necromass. **c** *E. coli* growth as an anoxic, monoculture and binary consortium based on qPCR analysis. Cell death occurred after 10–12 h of growth likely releasing necromass. **d** *C. phytofermentans* monoculture growth on different amino acid sources. No growth was observed from casamino acid-based medium nor on a nutritionally complete, chemically defined medium CSP which contained only free amino acids. Yeast extract (YE) contained peptides in addition to free amino acids. **e** *C. phytofermentans* monoculture growth on different concentrations of peptide-containing YE. **f** *C. phytofermentans* growth on different concentrations (High and Low) of *E. coli* necromass produced via sonication-induced lysis. Error bars represent the standard deviation from three biological replicates. See text for more details.

*E. coli* biomass yield on *C. phytofermentans* necromass was estimated with respect to two normalizations, cell number and cell mass. On a cell number basis, producing one *E. coli* cell required 2.0–2.2 cells of *C. phytofermentans* and on a mass basis, 1 g of *E. coli* biomass required 8.5–9.1 g of *C. phytofermentans* biomass. The differing values reflect the difference in *E. coli* and *C. phytofermentans* cell geometry and volume: *E. coli* cells are approximately 2 μm long while *C. phytofermentans* cells are approximately 10 μm long. The presented biomass yields and published biomass yields on necromass components suggest ~17–23% of the *C. phytofermentans* necromass was bioavailable for *E. coli*^41^. Free metabolite pools account for ~5% of cellular mass so some macromolecule degradation likely occurred^59,60^. This mechanism is believed to have played a large role in the enhanced productivity of the AOS grown biofilm cultures (Fig. 2e). Although, it is also proposed to play a role under anoxic conditions. CFU analyses suggested a large fraction of the *C. phytofermentans* culture formed spores during late exponential growth phase, lysing the vegetative cells, and releasing biomass components which would have been available for *E. coli* catabolism (Fig. 8b)^21,53^.

### Mechanism of enhanced consortium performance: catabolism of *E. coli* necromass

Catabolism of *E. coli* necromass by *C. phytofermentans* was explored; this was an additional mechanism for enhancing consortium productivity under anoxic conditions. *C. phytofermentans* cultures had a large increase in cell number when grown in a consortium, as compared to monoculture growth (Fig. 8a); the binary consortium had >10-fold more *C. phytofermentans* cells than the monoculture, based on qPCR. When the *C. phytofermentans* monocultures were analyzed using CFU analysis, the monocultures lost cell viability after 12 h of incubation with CFUs falling approximately 90% by 48 h of cultivation (Fig. 8b). However, the *C. phytofermentans* grown in consortia increased in CFUs until 24 h of incubation and retained >2.0 × 10^8^ CFU per biofilm at 48 h of cultivation (Fig. 8b). *E. coli* CFU counts decreased after exponential phase in both the monoculture and consortia experiments (Fig. 8c). Collectively, the results suggested resources from *E. coli*, potentially necromass, were promoting growth and sustaining viability of *C. phytofermentans*.

*E. coli* necromass was tested directly as a potential growth enhancer. *C. phytofermentans* did not grow on CSP chemically defined medium containing individual amino acids (Supplementary Table 2) nor did it grow on casamino acids, presumably requiring peptides supplied in the mGS-2 medium or from lysed cells (Fig. 8d). *C. phytofermentans* biomass accumulation increased with the addition of yeast extract which contained peptides along with other potential growth factors including trace metals and vitamins (Fig. 8e). *E. coli* necromass was generated by collecting biomass via centrifugation from mid-exponential phase, oxic monocultures. The biomass was washed twice with fresh mGS-2 medium and sonicated (Microson XL 2000) in an ice bath for 15 min at the maximum power setting to lyse the *E. coli* cells. The lysis solution was filtered using a 0.2 µm membrane to remove intact *E. coli* cells and the filtrate was used as a necromass source.

*C.* *phytofermentans* growth on *E. coli* necromass was evaluated under anoxic conditions as either a monoculture or binary consortium (Fig. 8b, f). *C. phytofermentans* monocultures had increased biomass accumulation which scaled with the addition of *E. coli* necromass (Fig. 8f). *C. phytofermentans* growth also increased when *E. coli* necromass was added to the binary consortium containing viable *E. coli* (Fig. 8b). The enhanced *C. phytofermentans* growth provides a basis for estimating the biomass yield of *C. phytofermentans* on *E. coli* necromass. On a cell number basis, one *C. phytofermentans* cell was produced from 16.5 to 19.1 cells of *E. coli* and on a mass basis,1 g of *C. phytofermentans* biomass was produced from 3.2 to 3.7 g of *E. coli* when added to mGS-2 medium. This figure assumed all *E. coli* cells were lysed and all necromass passed through the filter. This mechanism was likely responsible for the increased *C. phytofermentans* growth during both planktonic and biofilm growth.

## Discussion

**Fig. 9:**
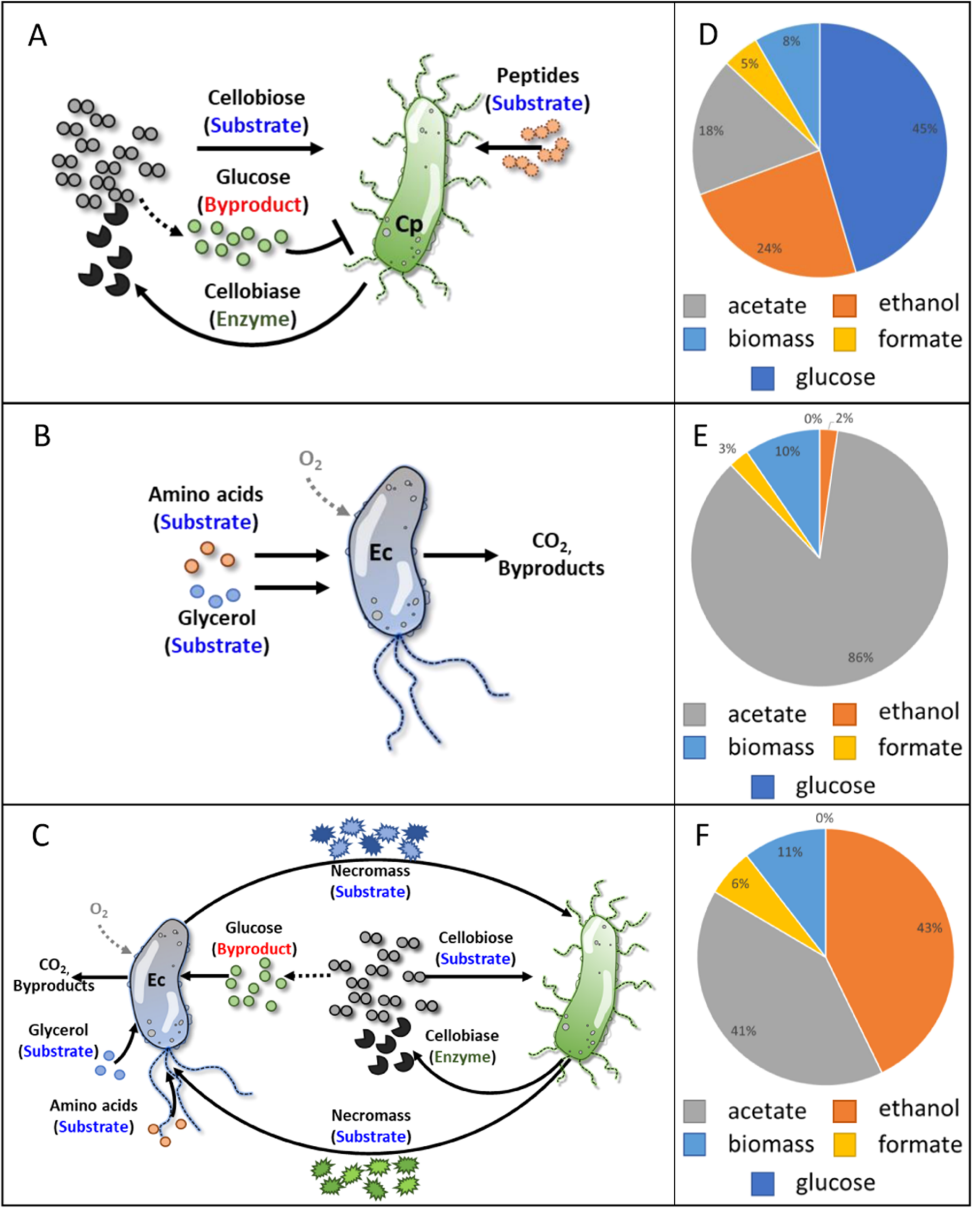
Proposed model of monoculture and consortia interactions and experimental distribution of reduced carbon products. **a** *C. phytofermentans* monoculture, **b** *E. coli* monoculture, **c** *C. phytofermentans*, and *E. coli* binary consortia with necromass catabolism. **d** Experimental distribution of carbon products for *C. phytofermentans* monoculture after 72 h of cultivation. Areas represent percent of measured carbon moles. **e** Experimental distribution of carbon products for anoxic *E. coli* monoculture after 72 h of cultivation. Areas represent percent of measured carbon moles. **d** Experimental distribution of carbon products for anoxic consortium after 72 h of cultivation. Areas represent percent of measured carbon moles.

An artificial consortium was assembled using principles identified in naturally occurring consortia including division of labor between primary- and secondary-resource specialists, metabolite exchange with positive feedback, and enhanced resource extraction based on necromass catabolism^27,41^. The cellobiose-degrading consortium comprised of *C. phytofermentans*, the primary resource specialist, and *E. coli*, the secondary-resource specialist, demonstrated the emergent properties of enhanced substrate depletion, enhanced ethanol secretion, and enhanced biomass productivity relative to the sum of monoculture properties. For example, the synergistic interactions improved planktonic and biofilm biomass productivity approximately 121% and 153%, respectively, on a mass basis (Table 1 and Fig. 2e). A proposed model of the monoculture and consortium substrate preferences and interactions is illustrated in Fig. 9a–c. Consortial interactions also produced substantial, experimental changes in byproduct distributions after 72 h of cultivation (Table 1 and Fig. 9d–f). The consortium used wild-type microorganisms to achieve the enhanced properties. Use of traditional metabolic engineering approaches such as deleting inefficient metabolic routes could further optimize the system as well as be used to synthesis other valuable bioproducts^39,40,61–63^.

Enhanced biomass productivity was proposed to be the result of a few major mechanisms. First, *C. phytofermentans* released cellobiase enzyme which hydrolyzed cellobiose into glucose extracellularly (Fig. 1g). The presence of free glucose inhibited the production of additional cellulolytic enzymes (Fig. 6a, b); when *E. coli* was present, it catabolized the glucose relieving inhibition of cellulolytic enzyme synthesis and created a positive feed forward loop enhancing the degradation of cellulose-derived sugar (Fig. 1g). In the presence of O_2_, *E. coli* likely catabolized fermentation byproducts removing the inhibitory metabolites, creating a positive feedback loop enhancing substrate catabolism. *C. phytofermentans* readily formed spores, lysing the vegetative cells, and releasing necromass which was partially bioavailable for *E. coli* catabolism (Figs. 7 and 8b). Additionally, the anoxic to oxic switch (AOS) cultivation would have lysed *C. phytofermentans* cells in the oxic zone of the biofilm, releasing necromass (Figs. 2 and 7). The spore-forming and O_2_-lysed cells would also release cellobiase which remained active in the presence of O_2_ (Fig. 6a, b), producing additional glucose for *E. coli* catabolism. Moreover, *E. coli* grew readily on simple substrates including free amino acids, upgrading those resources into proteins and oligomers; this upgrading combined with *E. coli* cell lysis would make the otherwise inaccessible resources available for the fastidious *C. phytofermentans*, enhancing its growth and production of cellulolytic enzymes (Fig. 8a, c).

The turnover of biomass from the primary resource population and the release of necromass is a common mechanism in natural consortia and can drive flux of material and energy between trophic levels^64–68^. Biomass turnover, through mechanisms like senescence, inhibitor-based cell lysis, or viral predation, can result in increased energy acquisition rates in the systems. This is a predictor of competitive consortium function based on a theory known as the “Maximum Power” principle^41,69,70^.

A substantial increase in biomass productivity occurred when the consortium was transferred from anoxic to oxic conditions. One hundred and forty-seven percent more consortia mass was produced during AOS cultivation as compared to anoxic cultivation (Fig. 2e). The increase was substantially larger (153%) than the sum of the monoculture AOS productivities, quantifying the outcome of the synergistic interactions between the two species and the oxic environment. The use of agar plates for biofilm cultivation prevented direct measurement of cellobiose utilization and ethanol production, but they are proposed to scale with biomass productivity suggesting >2-fold increase in cellobiose catabolism and ethanol production compared to monocultures. The AOS cultivation is a relatively simple strategy with a large impact and can be integrated into cultivation systems via the introduction of O_2_ after the initial anoxic phase. This strategy could be applied readily to either solid phase or heterogenous (liquid + flocs) bioreactors. The timing of the anoxic to oxic transition would need to account for the system growth rates, biomass concentration, and the length scales for O_2_ diffusion^71^.

The simultaneous use of both anaerobic and aerobic chemistries within the biofilm provides opportunity for bioprocessing. The anoxic zone would favor the capture of sugar-derived electrons on reduced products like ethanol, while the oxic zone enables high energetic yields on byproducts like acetate and high metabolic rates which consume O_2_ maintaining the anoxic zone. *E. coli* is a convenient biotechnological host and provides opportunities for producing a wide range of biochemicals in the anoxic, oxic, or both zones of the biofilm. Obligate aerobic or facultative *E. coli* strains could be cultivated in biofilms to control vertical localization, generating laminated catalytic potential^13,27^.

This study constructed an artifical *C. phytofermentans* and *E. coli* consortium based on biomimicry of naturally occuring, microorganism interactions. The consortium demonstrated the emergent properties of enhanced substrate depletion, enhanced ethanol production, and enhanced biomass productivity. The assembled consortium had enhanced functioning during both planktonic and biofilm cultivation based on crossfeeding, positive feedback mechanisms, and the catabolism of necromass. These design features are powerful tools for improving bioprocesses and can likely be incorporated within existing bioprocesses.

## Materials and methods

### Bacterial strains and medium

*C. phytofermentans* ISDg (ATCC 700394) and *E. coli* K-12 MG1655 were used for all experiments. All reported planktonic and biofilm growth were performed in modified GS-2 media^72^ (mGS-2) with the following composition per liter: 1.5 g KH_2_PO_4_, 2.9 g K_2_HPO_4_, 2.1 g urea, 10 g MOPS, 3.0 g Na-Citrate, 1 g Resazurin, 1 g yeast extract, 1 g MgCl_2_·6H_2_O, 150 mg CaCl_2_·2H_2_O, 1.25 mg FeSO_4_·6H_2_O, 2.3 g glycerol, 5 g cellobiose and 10 mL of trace metal solution (per liter: 1.5 g FeCl_2_·4H_2_O, 70 mg ZnCl_2_, 0.1 g MnCl_2_·4H_2_O, 6 mg H_3_BO_3_, 0.19 g CoCl_2_·6H_2_0, 2 mg CuCl_2_·2H_2_O, 24 mg NiCl_2_·H_2_O, 36 mg Na_2_MoO_4_·2H_2_O, 10 mL HCl (25%)). Salt solution (MgCl_2_·6H_2_O, CaCl_2_·2H_2_O, FeSO_4_·6H_2_O), cellobiose, yeast extract, and trace metal solution were sterilized separately by autoclave or filter sterilization and added after autoclaving. Initial pH of the basal components was adjusted to 6.9. When necessary, agar was added at 14 g L^−1^. Media was kept in an anaerobic chamber (Bactron II, Sheldon Manufacturing Inc.) with 5% H_2_, 5% CO_2_, and 90% N_2_ until it was used.

### Planktonic culturing

Planktonic experiments were performed using 18 × 150 mm Balch anaerobic culture tubes in containing 10 mL of mGS-2 medium in a shaker operated at 150 revolutions per minute and 37 °C. Initial cultures of each strain were prepared from cryogenically (−80 °C) frozen stock. Inocula were prepared from fresh overnight cultures grown in mGS-2 medium. Initial OD_600_ of each strain was 0.01 OD_600_ for *C. phytofermentans* and 0.001 for *E. coli* after dilution. Samples were collected aseptically using a 1 mL syringe to analyzed for OD_600_, pH, CFU, and extracellular metabolite concentration. Total sampling volume collected was less than 20% of initial culture volume. CFUs of *C. phytofermentans* and *E. coli* monoculture were determined using drop plating on agarose (1.5%) with mGS-2 media plates under anaerobic conditions. For consortium CFU counts, selective plates were used. *E. coli* counts were performed on mGS-2 agar plates cultured under oxic conditions to prevent *C. phytofermentans* growth. *C. phytofermentans* counts were performed on mGS-2 agar plates containing 50 µg mL^−1^ kanamycin to prevent *E. coli* growth.

Data analysis used the following conversion factors to quantify biomass: *E. coli*: 1 OD_600_ = 0.45 g cell dry weight L^−1^, 1 OD_600_ = 9.15 × 10^8^ CFU mL^−1^; *C. phytofermentans*: 1 OD_600_ = 1.55 g cell dry weight L^−1^, 1 OD_600_ = 7.55 × 10^8^ CFU mL^−1^. Parameters were either experimentally determined or from literature^73,74^.

### Colony biofilm culturing

Colony biofilm culturing systems consisted of 25 mm polycarbonate membrane disks with 0.22 µm pores (GVS Life Science, REF# 1215609) placed on mGS-2 agar plates^75–78^. Membranes were aseptically placed on mGS-2 agar plates and inoculated with 100 µL of planktonic cultures (0.01 OD_600_ for *C. phytofermentans* and 0.001 OD_600_ for *E. coli*). Biofilms were incubated at 37 °C in an anoxic chamber and/or oxic incubator depending on experiment. Biofilm cultures were aseptically transferred to a new medium plate every 2 days. Biofilm analysis was performed every 2 days using destructive sampling. Sampled colony biofilms were aseptically transferred to 5 mL of sterile phosphate-buffered saline (PBS) and vortexed vigorously for 30 s to separate cells from the membrane. The membrane was discarded, and the biofilm suspension was disaggregated using a high-performance dispersing instrument (T25 Ultra-Turrax, IKA) at 7000 revolutions per minute for 30 s. Further analysis (biomass, CFU and qPCR) was performed using this biofilm suspension.

### Extracellular metabolite analysis

Extracellular metabolite concentrations (glucose, acetate, lactate, ethanol, succinate, and formate) from planktonic cultures were measured using an Agilent 1200 HPLC. Samples were filtered with 0.2 µm centrifuge filter to remove cell debris. Twenty microliters of filtered samples were injected on an HPX-87H column (Bio-Rad) at 40 °C with a 0.005 M H_2_SO_4_ mobile phase (0.6 mL min^−1^). Data were collected with a refractive index detector and analyzed with Agilent ChemStation software.

### Spatial O_2_ concentrations within biofilms

Spatially resolved, in situ O_2_ concentrations were measured within biofilms using a MicroProfiling System from Unisense (Aarhus, Denmark). It consisted of a 25 µm O_2_ microsensor (OX-25), held by a motorized and computer-controlled micromanipulator (MM33-2) and microscope. The microsensor was calibrated with a strong reductant solution with both ascorbic acid and sodium hydroxide at a final concentration of 0.1 M and fully air saturated water with vigorous bubbling for 5 min. The O_2_ microsensor was positioned with the micromanipulator on the biofilm sample using a microscope. O_2_ gradients were measured every 25 µm from the top of the biofilm. Data were collected by SensorTrace Logger software from Unisense.

### Cryoprocessing of biofilms

Colony biofilms were cryoembedded using Tissue-Tek. Optimal cutting temperature (OCT) compound (Sakra Finetechnical Co.), dry ice, and a stainless steel slide for enhanced heat transfer. Vertical transections of biofilms were obtained by sectioning biofilms embedded in solidified OCT with a cryomicrotome. Thin section (10 µm) of vertical transects of the biofilms were placed onto polyethylene naphthalate (PEN) membrane-coated stainless microscope slide (Leica microsystems Inc.). The microscope slides were kept at −20 °C until analysis.

### Laser microdissection (LMD) of biofilm

Leica LMD6 (Leica microsystems Inc.) was used to dissect and capture sections from different regions within the biofilm. PEN membrane microscope slides containing biofilm were examined using lenses with objectives of ×10 to ×40 magnification. Samples were obtained using the laser cut and capture sequence which allow dissected samples to be captured into 20 µl of enzymatic lysis buffer (20 mM Tris-Cl, 2 mM sodium EDTA, 1.2% Triton X-100, 20 mg mL^−1^ lysozyme at pH 8.0). Samples were collected from three vertical positions (top, middle, bottom) at four to six different radial positions from a single biofilm.

### qPCR analysis of species abundance and distribution

qPCR was performed to analyze species abundance in both planktonic and biofilm cultures. DNA was extracted and processed with DNeasy Kit or DNeasy Micro kit (Qiagen) using the manufacturer protocols. DNA samples were stored at −20 °C until analysis by qPCR. Primers for 16s rRNA genes (Table 3) were evaluated in silico using IDT Oligoanalyzer tool and NCBI’s primer Blast tool. Additionally, primer independency between *C. phytofermentans* and *E. coli* was confirmed both by 16s RNA sequence alignment using Mega7 software and by experimental testing using Rotor-Gene 3000 (Corbett Research) with QuantiFast SYBR Green PCR Kit (Qiagen). Genomic DNA from *C. phytofermentans* and *E. coli* monocultures were extracted and quantified with Qubit Fluorometer (Thermo Fisher) and used to create a standard DNA curve for each species. Cycling parameters were as follows: PCR initial heat activation at 95 °C for 5 min, 40 cycles of 95 °C for 10 s and 60 °C for 30 s. Data were acquired during 60 °C analyzing step and calculated threshold cycle (*C*_T_) values with Roto-Gene6 software. Equation (1) was used to calculate the DNA copy number for each species^79,80^:

1 $${\mathrm {DNA}}\,{\mathrm {copy}}\,{\mathrm {number}} = \frac{{6.02 \times 10^{23}\left({\mathrm{copy}}\,{\mathrm {per}}\,{\mathrm {mol}} \right) \times {\mathrm {DNA}}\,{\mathrm {amount}}\,({\mathrm {g}})}}{{{\mathrm {DNA}}\,{\mathrm {length}}\,\left( {{\mathrm {bp}}} \right) \times 660\,({\mathrm {g}}\,{\mathrm {per}}\,({\mathrm {mol}} \times {\mathrm {bp}}))}}.$$

**Table 3:** Primer sequences for *E. coli* and *C. phytofermentans* 16s rRNA genes.

Strain	Primer	Sequence	Conc. (nM)	*T* (°C)
*E. coli*	16s rRNA-For 16s rRNA-Rev	ACG TTA CCC GCA GAA GAA GC TTC CGA TTA ACG CTT GCA CC	250 250	58 56
*C. phytofermentans*	16s rRNA-For 16s rRNA-Rev	ACA GGG GGA TAA CAG TCG GA TCG CCT TGG TAG GCC ATT AC	250 250	58 57

Primer sequences were analyzed using Mega7 genetic analysis tool to confirm uniqueness and independency between species. Primer independency was confirmed with experimental testing of culture samples.

DNA copy number was divided by the 16s rRNA copy number per chromosome (*E. coli*: 7 copies, *C. phytofermentans*: 8 copies) to calculate the total cell equivalents. Calibration curves can be found in Supplementary Fig. 6. The cell number could be converted to other quantities such as OD_600_, CFU L^−1^, and g cell dry weight L^−1^ using through conversion factors listed in the “Planktonic culturing” section.

### Reporting summary

Further information on research design is available in the Nature Research Reporting Summary linked to this article.

## Supplementary information

Supplementary Information

Reporting Summary

## Data Availability

The data that support the findings of this study are available from the corresponding author upon reasonable request.
