# Supplementary material for: Artificial consortium demonstrates emergent properties of enhanced cellulosic-sugar degradation and biofuel synthesis
Source: NPJ Biofilms Microbiomes. 2020 Dec 2;6:59. doi: 10.1038/s41522-020-00170-8 (PMC7710750; doi:10.1038/s41522-020-00170-8)
Supplement: Supplementary file 1 — Supplementary Information [file 41522_2020_170_MOESM1_ESM.pdf]

Supplementary Material

Title: Artificial consortium demonstrates emergent properties of enhanced cellulosic-sugar degradation and biofuel synthesis

Authors: Heejoon Park[1,2], Ayushi Patel,[3] Kristopher A Hunt[1,2ǂ], Michael A. Henson,[3] Ross P. Carlson[1,2]*

Affiliations:

1. Department of Chemical and Biological Engineering, Montana State University, Bozeman, Montana, USA
2. Center for Biofilm Engineering, Montana State University, Bozeman, Montana, USA
3. Department of Chemical Engineering and Institute for Applied Life Sciences, University of Massachusetts, Amherst, Massachusetts, USA

ǂ current address: Department of Civil and Environmental Engineering, University of Washington, Seattle, Washington, USA

*Corresponding author: Ross P. Carlson, rossc@montana.edu

Supplementary Table 1. Summary growth properties for *E. coli* (Ec) and *C. phytofermentans* (Cp) monocultures and binary consortium (EcCp) grown on mGS-2 medium with and without glycerol.

	Ethanol (mM)			Formate (mM)			Acetate (mM)		
	Ec	Cp	EcCp	Ec	Cp	EcCp	Ec	Cp	EcCp
**w/ gly**	1.24	3.27	26.71	1.00	1.27	7.49	16.79	2.41	25.40
**wo/gly**	0.46	3.05	20.16	0.00	0.49	4.70	7.36	1.38	19.18

**EtOH/For (mol/mol)**	Ec	Cp	EcCp
**w/ gly**	1.25	2.58	3.57
**wo/gly**	N/A	6.17	4.29

**EtOH/Ace (mol/mol)**	Ec	Cp	EcCp
**w/ gly**	0.07	1.36	1.05
**wo/gly**	0.06	2.21	1.05

Supplementary Table 2. Chemically-defined CSP medium.

4 g/L glucose
0.7 g/L sodium citrate
0.1 g/L EDTA tetrasodium salt
1.7 g/L yeast nitrogen base
1X minimum essential media (MEM) non-essential amino acid (100X solution)
1X minimum essential media (MEM) amino acid (50X solution)
4.7 g/L KH_2PO_4
8.2 g/L Na_2HPO_4
0.02 g/L adenine
0.02 g/L uracil
0.02 g/L cytosine
0.02 g/L guanine
0.15 g/L glutamine
2.0×10^{-6} g/L vitamin B_{12}
2.8×10^{-3} g/L $FeSO_4 \cdot 7H_2O$
1.2×10^{-5} g/L $CoCl_2 \cdot 6H_2O$

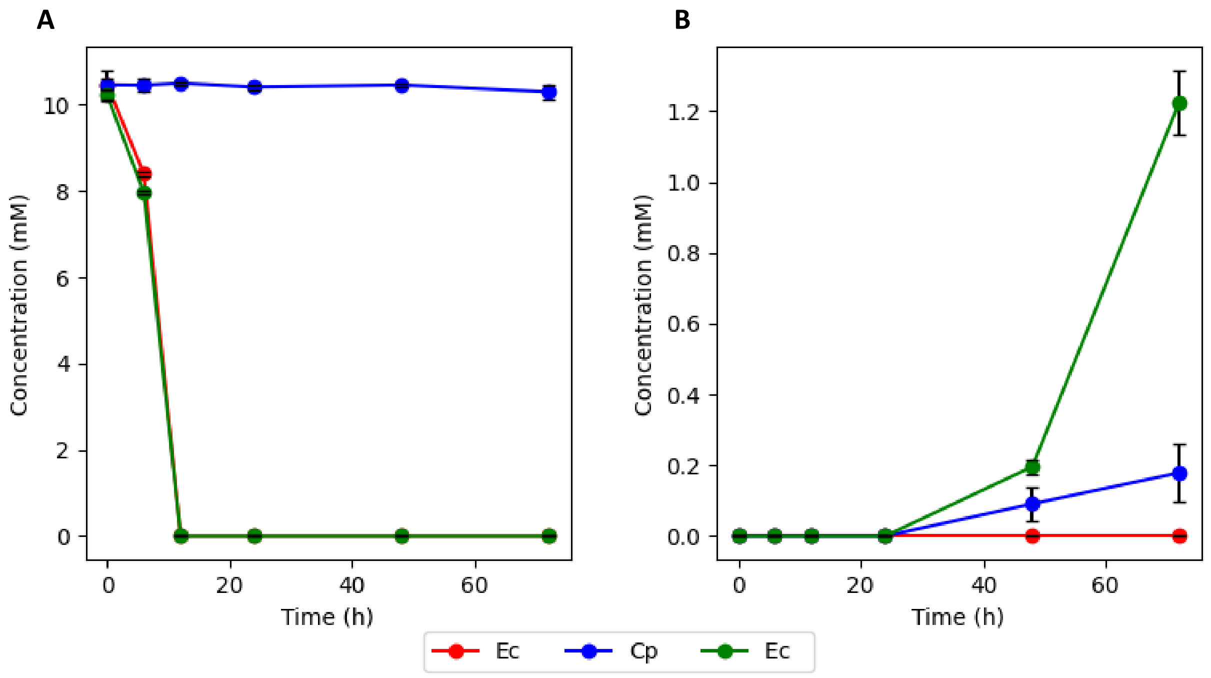

Supplementary Figure 1. Planktonic growth properties for *E. coli* (Ec) and *C. phytofermentans* (Cp) monocultures and a binary consortium (EcCp) grown anoxically on mGS-2 medium containing glycerol. (**A**) Citrate concentration. (**B**) Lactate concentration. Error bars represent standard deviation from three biological replicates. See Figure 1 in the main text for additional data.

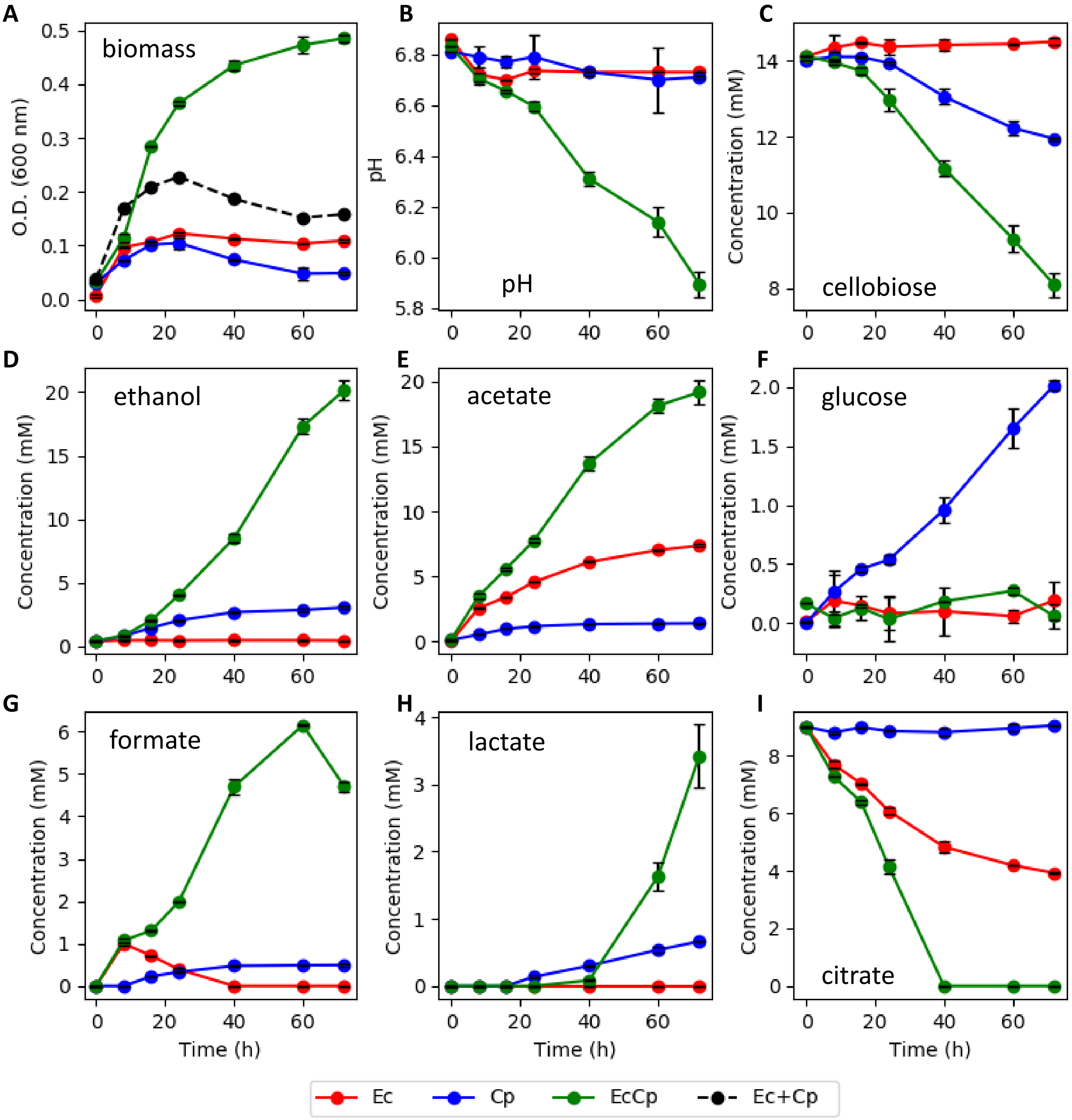

Supplementary Figure 2. Planktonic growth properties for *E. coli* (Ec) and *C. phytofermentans* (Cp) monocultures and a binary consortium (EcCp) grown on mGS-2 medium without glycerol. (**A**) Optical density (O.D.$_{600}$), (**B**) pH, (**C**) cellobiose concentration, (**D**) ethanol concentration, (**E**) acetate concentration, (**F**) glucose concentration, **(G)** formate concentration, **(H)** lactate concentration, and **(I)** citrate concentration. Ec+Cp indicates the sum of *E. coli* and *C. phytofermentans* monoculture properties. Error bars represent the standard deviation from three biological replicates.

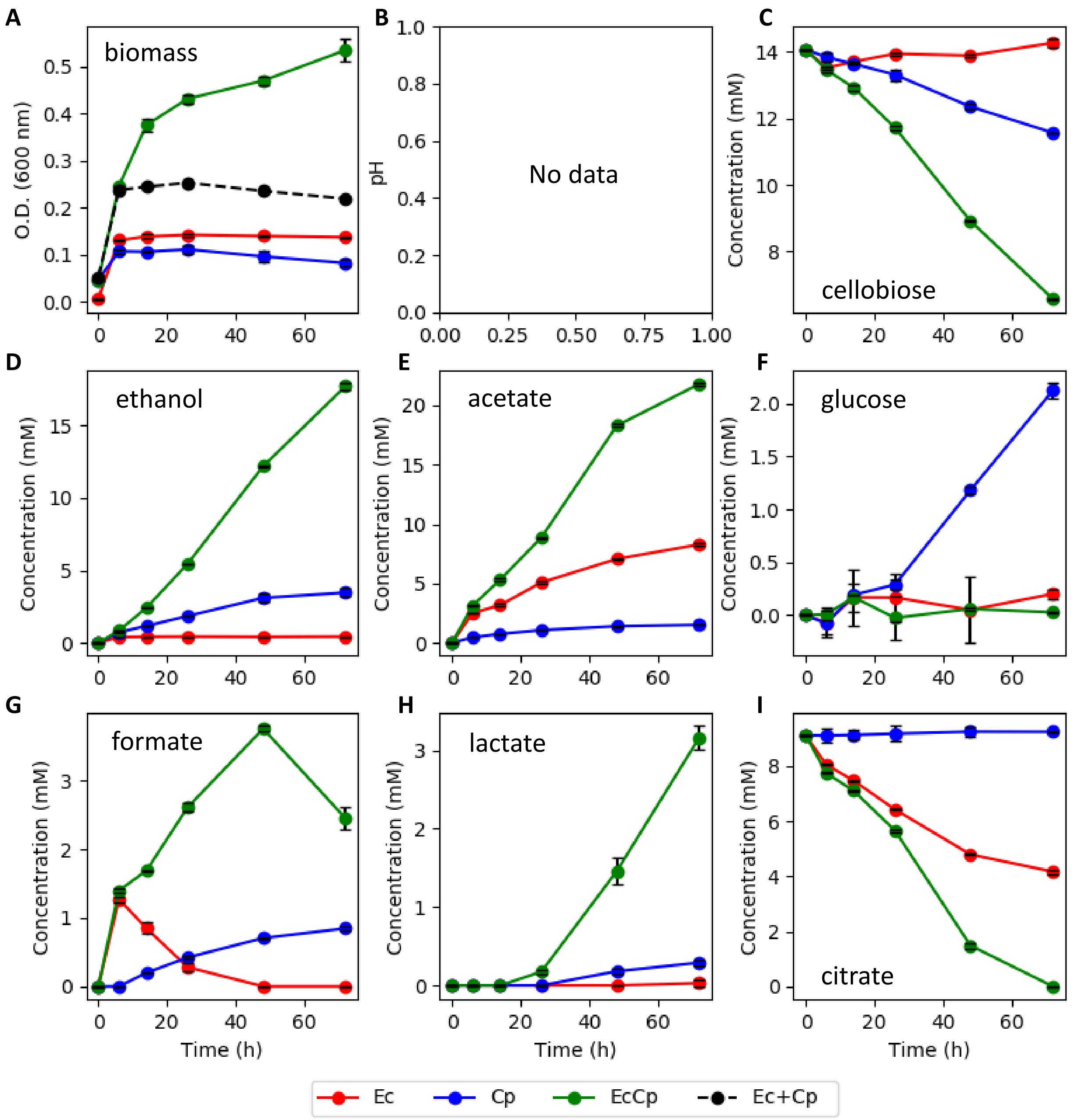

Supplementary Figure 3. Planktonic growth properties for *E. coli* (Ec) and *C. phytofermentans* (Cp) monocultures and a binary consortium (EcCp) grown on mGS-2 medium without glycerol but with supplements of nitrogen and sulfur sources ($MgSO_4$ =10 mM, NH_4CL = 10 mM.) (**A**) Optical density (O.D.$_{600}$), (**B**) pH was not measured for this condition, (**C**) cellobiose concentration, (**D**) ethanol concentration, (**E**) acetate concentration, (**F**) glucose concentration, **(G)** formate concentration, **(H)** lactate concentration, and **(I)** citrate concentration . Ec+Cp indicates the sum of *E. coli* and *C. phytofermentans* monoculture properties. Error bars represent the standard deviation from three biological replicates.

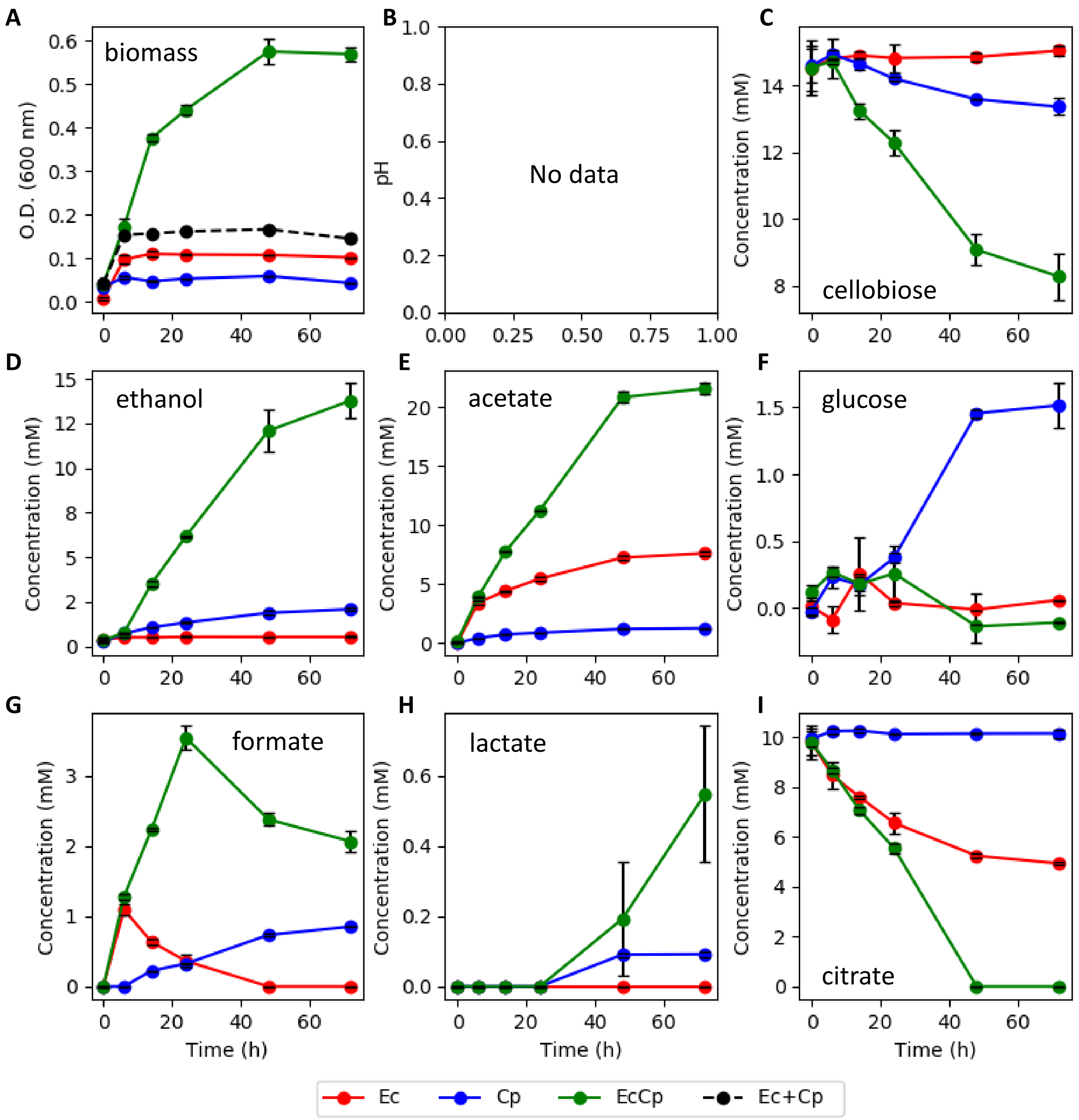

Supplementary Figure 4. Planktonic growth properties for *E. coli* (Ec) and *C. phytofermentans* (Cp) monocultures and a binary consortium (EcCp) grown on mGS-2 medium without glycerol and without urea (**A**) Optical density (O.D.$_{600}$), (**B**) pH was not measured for this condition, (**C**) cellobiose concentration, (**D**) ethanol concentration, (**E**) acetate concentration, (**F**) glucose concentration, **(G)** formate concentration, **(H)** lactate concentration, and **(I)** citrate concentration. Ec+Cp indicates the sum of *E. coli* and *C. phytofermentans* monoculture properties. Error bars represent the standard deviation from three biological replicates.

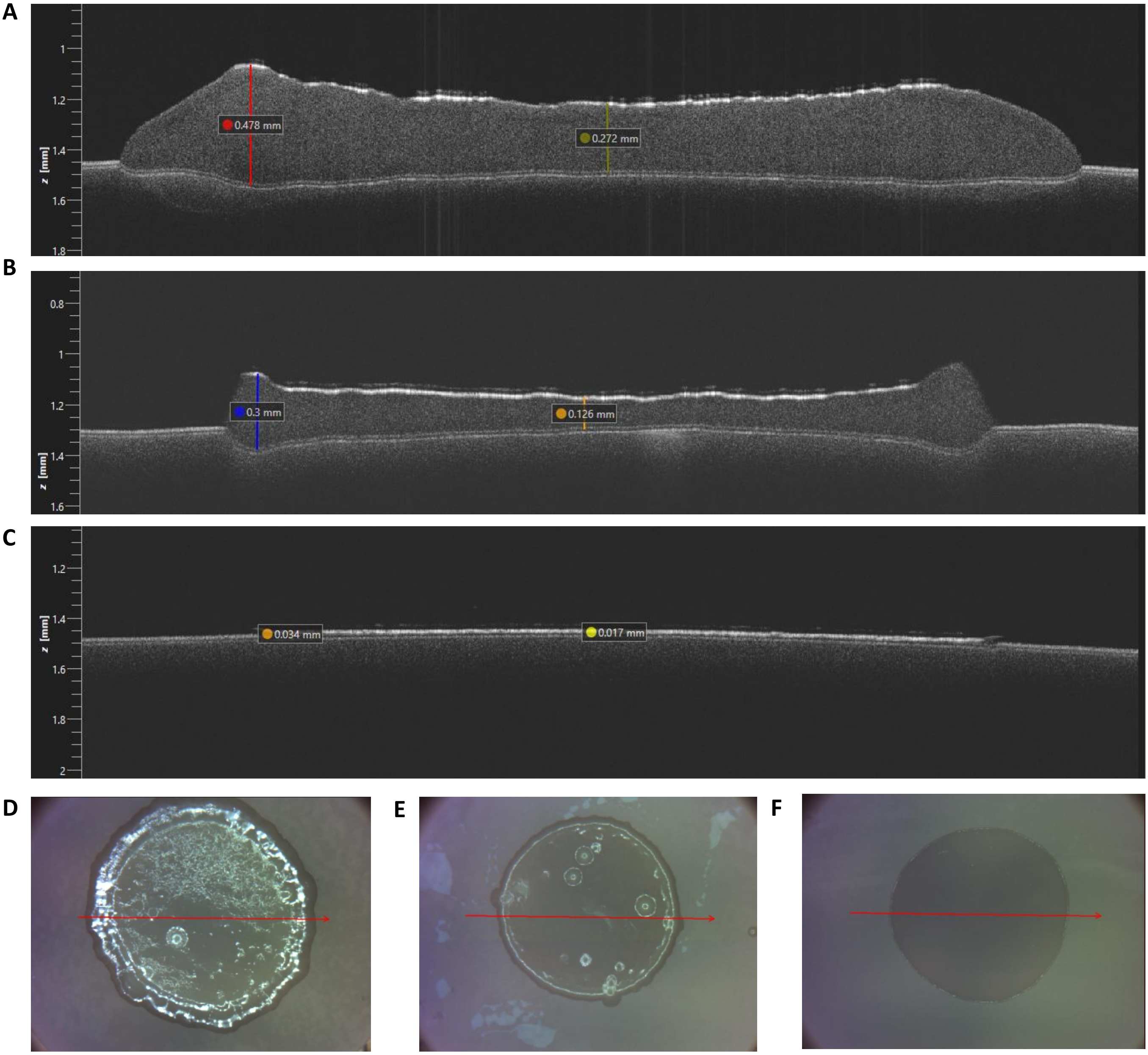

Supplementary Figure 5. Optical coherence tomography (OCT) analysis of coculture biofilm comprised of *E. coli* (Ec) and *C. phytofermentans* (Cp) grown under different conditions. **(A)** and **(D)** anoxic to oxic shift (AOS) growth conditions, **(B)** and **(E)** anoxic (AN) growth conditions, and **(C)** and **(F)** oxic (OX) growth conditions. Analysis performed after 10 days of cultivation.

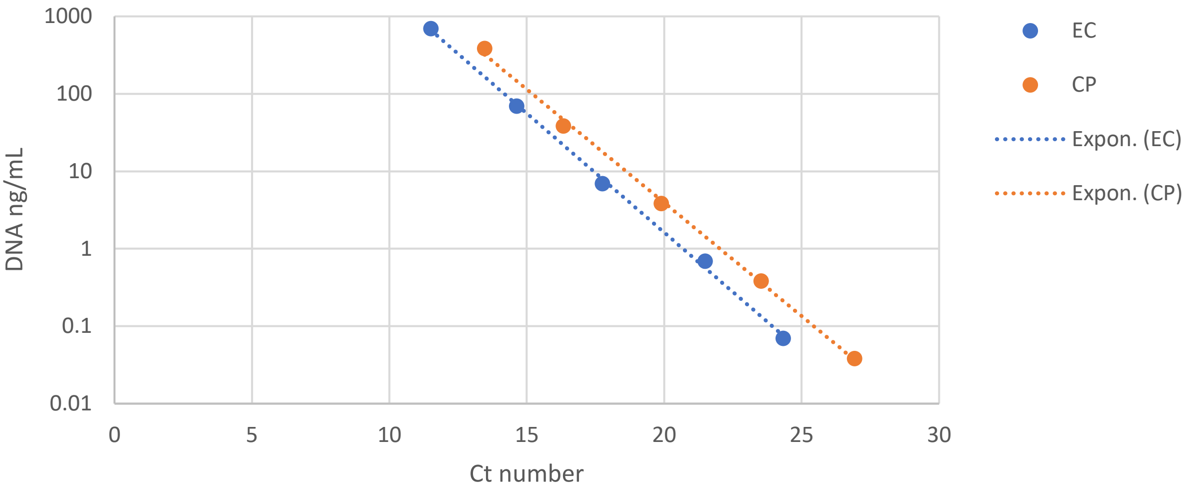

E.coli	*C. phytofermentans*
$y = 2E{+}06e^{-0.708x}$ $R^2 = 0.9987$	$y = 3E{+}06e^{-0.674x}$ $R^2 = 0.9985$

Supplementary Figure 6. qPCR calibration curves for relationship between DNA mass (ng/ml) and Ct number for *E. coli* and *C. phytofermentans* cultures. DNA mass was quantified using Qubit analysis.
